# Bursty Communication Patterns Facilitate Spreading in a Threshold-Based Epidemic Dynamics

**DOI:** 10.1371/journal.pone.0068629

**Published:** 2013-07-19

**Authors:** Taro Takaguchi, Naoki Masuda, Petter Holme

**Affiliations:** 1 Department of Mathematical Informatics, The University of Tokyo, Tokyo, Japan; 2 IceLab, Department of Physics, Umeå University, Umeå, Sweden; 3 Department of Energy Science, Sungkyunkwan University, Suwon, Korea; 4 Department of Sociology, Stockholm University, Stockholm, Sweden; Centre de Physique Théorique, France

## Abstract

Records of social interactions provide us with new sources of data for understanding how interaction patterns affect collective dynamics. Such human activity patterns are often bursty, *i.e.*, they consist of short periods of intense activity followed by long periods of silence. This burstiness has been shown to affect spreading phenomena; it accelerates epidemic spreading in some cases and slows it down in other cases. We investigate a model of history-dependent contagion. In our model, repeated interactions between susceptible and infected individuals in a short period of time is needed for a susceptible individual to contract infection. We carry out numerical simulations on real temporal network data to find that bursty activity patterns facilitate epidemic spreading in our model.

## Introduction

Communication between individuals is a fundament of human society. Nowadays technologies such as sensor devices and online communication services provide us with records of interaction between individuals, including face-to-face conversations, e-mail exchanges, and phone calls, in massive amounts. Such data often consist of a sequence of interaction events. Each event is represented by a triplet, *i.e.*, the IDs of two individuals involved in the event and the time of the event. One traditional way to characterize such data is to represent them as an aggregated network, in which the links are drawn between two nodes (*i.e.*, individuals) that communicate in at least one event, and investigate structural properties of the aggregated static networks [1]. Another and richer representation of this type of data is to model them as temporal networks, in which the links between two nodes exist only at the time of an event [2].

Effects of temporal networks on contagious phenomena, such as infectious diseases and rumors, have been investigated by various authors. To simulate spreading dynamics on temporal networks, we read the events in an empirical event sequence one by one in the chronological order and possibly update the states (*e.g.*, susceptible and infected) of the two nodes involved in the event. Karsai and colleagues simulated the susceptible-infected (SI) model on temporal networks and found that bursty activity patterns slow down contagions [3]; Bursty activity patterns are identified with a long-tailed distribution of the interevent times (IETs) [4], [5]. The slowing down occurs because, at an arbitrary time point, the average time to the next event is longer for the long-tailed IET distribution than for the exponential IET distribution with the same mean. In other words, after an individual gets infected, it tends to take longer time to infect the neighbors under the long-tailed as compared to exponential IET distribution. Other numerical [6], [7] and analytical [8]–[10] results also support that the long-tailed IET distribution mitigates contagion. However, the burstiness was reported to accelerate contagion on a different data set [11] and a different type of epidemic dynamics. Our understanding of the effect of the burstiness, and other temporal structures, on contagious processes is still elusive.

In the present study, we show that bursty activity patterns facilitate epidemic spreading in a variant of the deterministic threshold model [12], [13]. In standard models of epidemics including the SI, susceptible-infected-recovered (SIR), and susceptible-exposed-infected-recovered (SEIR) models, which have been employed in the literature cited above, a susceptible node gets infected from an infected neighbor with a constant probability in an event, regardless of the amount of exposure to infected neighbors in the past. However, history-dependent thresholding effects in which the thresholding operates on the concentration of the pathogen have been reported for some infectious diseases mediated by bacteria, such as the tuberculosis and the dysentery [14]. In the case of information propagation, the exposure to the information increases one's interest in a topic, and the attractiveness of a topic decays in time in the absence of stimulus [15], [16]. We may need multiple interactions to persuade others to do something, and repeated contacts in a short period can be more effective than those dispersed over a long period. In general, contacts with the same person need not be as influential as the same number of contacts with different persons. In this work, we do not model such effects but focus on the limit where contacts are worth equally much. To consider this type of infection, we generalize the deterministic threshold model to the case of history dependence and memory decay and simulate the proposed model on temporal network data.

## Results

### Simulation protocols and data sets

Each node 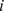 is assumed to have an internal variable denoted by 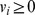 (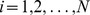), which represents, for example, the concentration of a pathogen in the individual or the individual's interest in a topic. Initially, 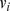 to equal to zero for all 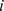. We assume that node 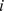 is in the susceptible (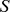) state before 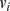 exceeds a threshold value 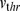 and that node 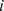 is in the infected (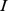) state once 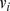 exceeds 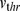. Each node is in either state. Nodes in state 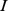 never return to state 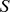; our model is an extension of the SI model. Therefore, the number of 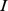 nodes monotonically increases in time. It should be noted that the SI model was used in place of the more realistic SIR model in previous literature on static [17] and temporal [3], [11] networks. This is because the initial growth phase, which determines the possibility of large-scale spreading initiating from a small number of infected individuals, is the same between the SI and SIR models.

**Figure 1:**
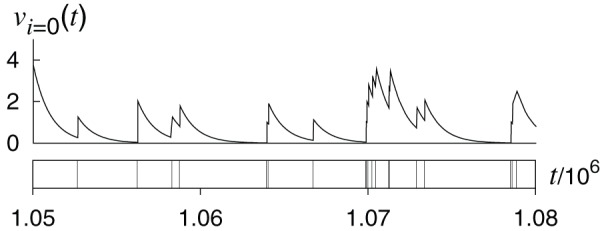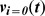 for 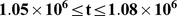 in Email data set. We set 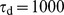. The vertical ticks in the box plot in the bottom indicate the times of the events that involve node 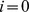.

When node 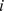 in state 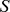 interacts with an 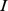 node through an event, 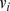 is increased by unity. In the absence of interaction with 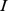 nodes, 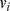 is assumed to decay exponentially in time. In other words, 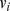 is given by
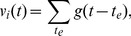
(1)where
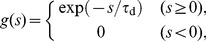
(2)and 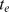 is the time of an event between node 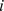 and an 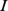 node, and 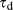 is the decay time constant. An example time course of 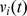 is shown in Figure 1.

The model contains two parameters 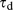 and 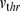 and can be regarded as a variant of the deterministic threshold model [12], [13]. Although we assume that all the nodes have the same values of 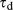 and 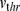 for simplicity, it is straightforward to generalize the model in the case of heterogeneous parameter values.

**Figure 2:**
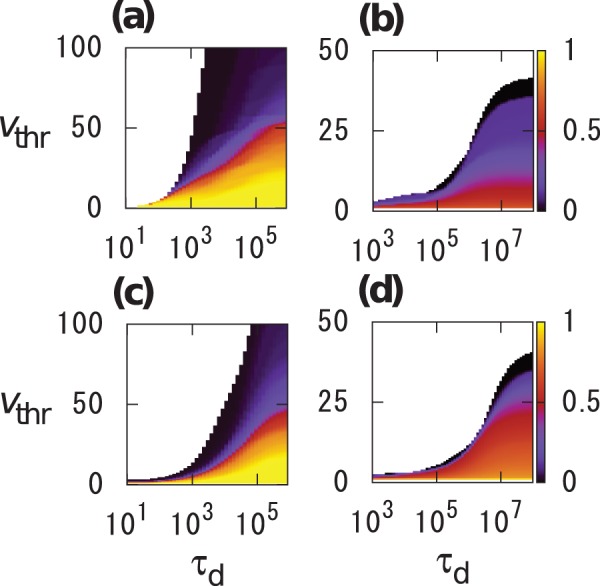
Dependence of the final infection size 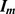 on 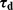 and 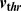. (a), (b) Original temporal networks. (c), (d) Randomized temporal networks. (a), (c) 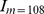 in Conference data set. (b), (d) 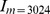 in Email data set. No infection occurs in the blank parameter regions. The parameter values for which at least one infection occurs are colored.

We simulate our model numerically on empirical temporal networks in the following way. At 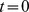, we select a node as initial seed 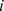 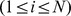 and set its state to 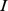. All the other nodes are initially in state 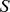. Then, we chronologically read the event sequence one by one and update 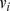 and the states of the two nodes involved in the event. Because our model is deterministic, the final infection size (*i.e.*, fraction of 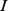 nodes at time 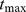, where 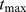 is the time of the last event in the data set), denoted by 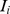, is unique for given initial seed 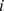, 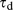, and 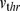. We examine spreading dynamics starting from all the possible initial seeds, except for the results shown in Figure 2 for which we select the node with the maximum number of events as the seed.

We use two data sets. The first data set, called Conference in the following, is the face-to-face conversation log between attendees of a scientific conference [18]. The second data set, called Email, is the record of e-mail exchanges between the members of a university [19]. In the second data set, we neglect the direction of the interaction (*i.e.*, from sender to receiver) for simplicity. The basic statistics of the data sets are summarized in Table 1.

**Table 1:** Statistics of the two data sets.

	Conference	Email
Number of nodes (*N*)	113	3,188
Number of events	20,808	309,125
Recording period	3 days	83 days
Time resolution	20 sec	1 sec

### Effects of the burstiness on infection size

In Figures 2(a) and 2(b), we plot the dependence of final infection size 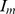 on 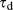 and 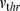 for initial seed node 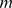 having the maximum number of events in Conference and Email data sets, respectively. In the blank parameter region, no infection occurs such that 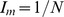. Naturally, 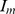 increases with 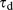 and decreases with 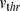.

Next, we carry out the same set of simulations on the randomized temporal networks for the sake of comparison. To this end, we use the so-called randomly-permuted-times randomization, in which the time stamps of all the events are randomly shuffled [2], [3], [6]. The randomization eliminates temporal properties of the original temporal networks such as bursty activity patterns and the pairwise correlations of the IETs, whereas it conserves all the properties of the aggregated networks, *i.e.*, weighted adjacency matrix. In addition, daily and weekly activity patterns are conserved at the population level although they are not conserved for each individual.

For the randomized temporal networks, the dependence of 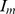 on 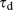 and 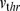 are shown in Figures 2(c) and 2(d) for Conference and Email data sets, respectively. We find that the parameter region in which infection occurs is larger for the original temporal networks (colored regions in Figures 2(a) and 2(b)) than for the randomized temporal networks (colored regions in Figures 2(c) and 2(d)) for intermediate values of 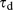 (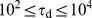 and 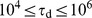 for Conference and Email data sets, respectively). In the original data sets, the nodes tend to have many events in bursty periods and be quiescent in other periods. The randomization procedure eliminates bursty activity patterns. Therefore, 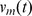 can reach 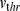 in such a bursty period for the original but not randomized temporal networks if 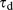 and 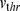 take intermediate values. In the randomized data sets, 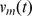 tends to decay faster than it grows, although the number of events per node is the same between the original and randomized data.

For Email data set, 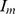 for the randomized data set (Figure 2(d)) is larger than that for the original data set (Figure 2(b)) when 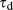 is large and 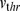 is small. This is mainly because the randomization considerably increases the reachability ratio of initial seed 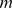. The reachability ratio of a node is defined as the fraction of nodes that we can reach from the node by tracing the events in the chronological order [20]. If every event can elicit infection, which is the case when 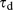 is large and 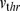 is small, 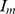 is approximated by the reachability ratio of node 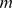. The reachability ratio of node 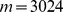 in Email data set is equal to 0.7458 and 0.9981 for the original and randomized data sets, respectively. In contrast, the reachability ratio of node 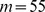 in Conference data set is equal to 0.9642 and 1 for the original and randomized data sets, respectively; the difference is smaller than in the case of Email data set.

**Figure 3:**
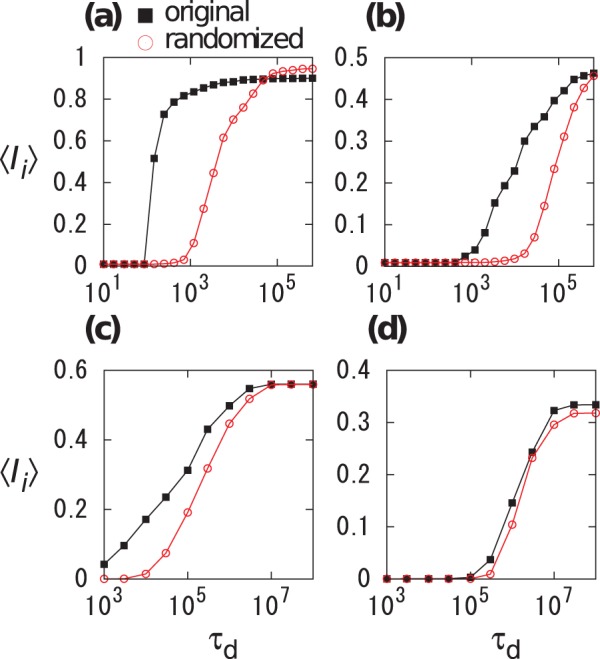
Average final infection size 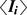 for (a), (b) Conference and (c), (d) Email data sets. Squares and circles correspond to the original and randomized temporal networks, respectively. We set (a) 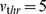, (b) 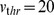, (c) 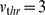, and (d) 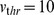.

In Figure 3, the average final infection size 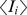, defined as the average of 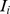 over all the nodes 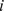, is plotted as a function of 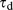 for two values of 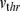 for each data set. Figure 3 indicates that 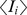 for the original temporal networks is larger than that for the randomized temporal networks for a broad range of 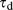 for both data sets. For Email data set, the 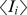 values for the original data set are similar to or larger than those for the randomized data set, even for large 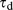 (Figures 3(c) and 3(d)). These results are apparently inconsistent with the fact that 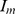 is larger for the randomized data set than for the original data set (Figures 2(b) and 2(d)) for large 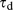. The inconsistency may be caused by the competition between two opposite effects of the randomization. First, the randomization tends to increase the reachability ratio of each node to enhance epidemic spreading. Second, the randomization eliminates the burstiness to suppress epidemic spreading. For nodes involved in many events, the first effect would dominate the second effect (Figures 2(b) and 2(d)) and vice versa for nodes involved in a small number of events. We will also discuss this point in Discussion.

**Figure 4:**
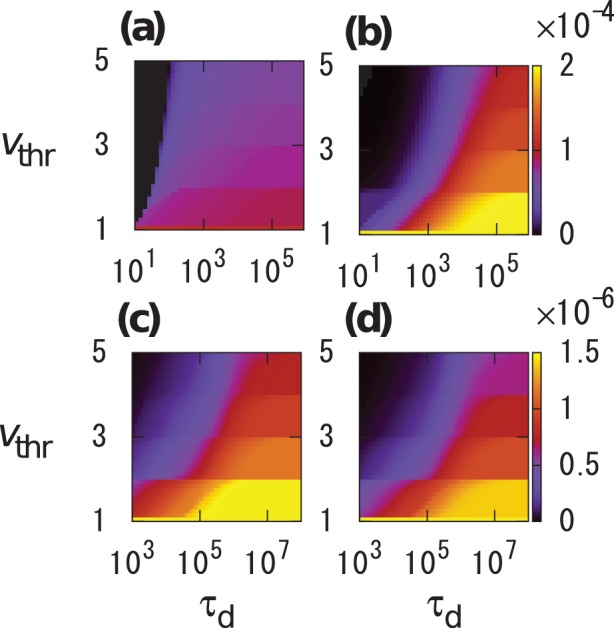
Average single-link infection rate 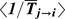 for (a), (b) Conference and (c), (d) Email data sets. (a), (c) Original temporal networks. (b), (d) Randomized temporal networks.

In the bond percolation on static networks, the probability that single bonds are open (independent of different bonds) is the sole parameter that determines the possibility that the entire network has a giant component [1]. Motivated by this picture, we hypothesize that the results shown in Figures 2 and 3 are largely explained by the bursty nature of events on single links. In other words, we speculate that the structure of the aggregated networks or correlation between event sequences on different links do not much influence the results. To test the hypothesis, we separately examine the event sequence on each link. For each link, *i.e.*, node pair 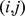 with at least one event, 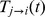 is defined as the time required for node 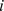 to be infected since node 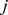 is infected at time 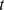. We emphasize that we do not consider influences from other nodes on 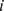 in this analysis. We take the time average of 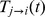, denoted by 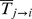, over 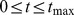. A problem with the time averaging is that 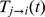 is indefinite for sufficiently large 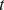 because 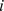 does not get infected by time 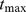. Therefore, we adopt the boundary condition in which the first events between nodes 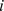 and 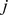 virtually replay after 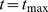. We denote the time of the first event between 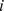 and 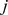 by 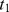. If we temporarily set 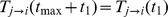, it takes at most 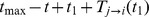 for node 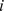 starting with 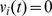 to be infected from node 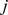, where 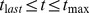 and 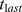 is the last time before which 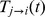 is finite. Therefore, we set 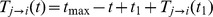 for 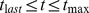. This boundary condition is the same as that is used in Ref. [21] for defining the average temporal path length. If 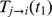 is indefinite (*i.e.*, infection never occurs between 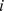 and 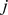), 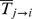 is set to infinite. We define denoted by 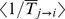 as the average of 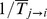 over the 20% links with the largest numbers of events, because the majority of the links possesses a small number of events in both data sets. This thresholding leaves 441 and 6,932 links for Conference and Email data sets, respectively.


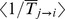
 for the original and randomized temporal networks are shown for various 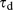 and 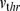 values for Conference (Figures 4(a) and 4(b)) and Email (Figures 4(c) and 4(d)) data sets. Because infection can be induced only through a single link in the present simulations, we examined 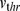 values that are much smaller than those used in Figures 2 and 3. For both data sets, 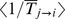 for the original temporal networks (Figures 4(a) and 4(c)) is larger than that for the randomized networks (Figures 4(b) and 4(d)) for intermediate values of 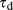 (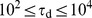 and 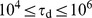 for Conference and Email data sets, respectively). The behavior of 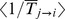 is consistent with the results of the network-based simulations (Figures 2 and 3).

## Discussion

We numerically simulated a variant of the deterministic threshold model on empirical temporal networks. We found that the average final infection size for the empirical temporal networks is larger than those for the randomized temporal networks in a broad parameter region (Figures 2 and 3). The bursty nature of the IETs on single links has a sufficient explanatory power for the results of the network-based simulations (Figure 4). The burstiness promoted epidemic spreading when the decay exponent 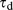 takes an intermediate value (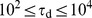 and 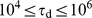 (seconds) for Conference and Email data sets, respectively). This range of 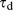 may be practical because the influence of a pathogen that an individual has received may last for hours to days.

The finding that the burstiness facilitates the spreading also sheds light on a function of the redundant interaction events. We previously found that about 80% of the events are redundant in the sense that they affect little on bridging efficient temporal paths in Conference data set [22]. However, for the spreading dynamics in our model, such redundant events play a crucial role in increasing 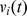 within bursty periods. Threshold models can be generalized to temporal networks in several ways. Reference [23], for example, considers a sliding window where only contacts within the window matters for the spreading. The authors examined two types of threshold rules–whether the threshold operates on all the total number of contacts with I in the interval or on the fraction of such contacts. The output was data dependent, but for most of their datasets, the results for the present model are similar to their results in the case of an absolute threshold. This suggests that we should be careful in generalizing our results too much (even though they should probably hold true for email and conference contacts).

In the previous section, we mentioned two possible consequences of the randomization of temporal networks: weakened burstiness and enhanced reachability. To disentangle the contribution of the two factors to epidemic spreading is difficult, because the two factors are simultaneously affected by the present randomization scheme. Therefore, looking for alternative randomization procedures or generative models of temporal networks in which burstiness and reachability are independently controlled is warranted for future work.
